# Effects of Fertility-Limiting Hormones Quinestrol and Levonorgestrel on Bait Intake and Reproductive Performance of the Nile Grass Rat (*Arvicanthis niloticus* Rüppel, 1842), a Major Agricultural Rodent Pest in Africa

**DOI:** 10.3390/ani16142134

**Published:** 2026-07-09

**Authors:** Daniel Desta, Abadi Berhe, Dawit Kidane, Kiros-Meles Ayimut, Kiros Welegerima, Apia W. Massawe, Rhodes H. Makundi, Steven R. Belmain, Yonas Meheretu

**Affiliations:** 1 Department of Biodiversity and Ecotourism Management, Mekelle University, Mekelle P.O. Box 231, Ethiopia; daniel.desta@mu.edu.et (D.D.); abadi.berhe1@gmail.com (A.B.); 2 Department of Biology, Mekelle University, Mekelle P.O. Box 231, Ethiopia; dawit.kidane@mu.edu.et (D.K.); kiros.welegerima@mu.edu.et (K.W.); 3 Department of Dryland Crop and Horticultural Sciences, Mekelle University, Mekelle P.O. Box 231, Ethiopia; kirosm62@gmail.com; 4 Institute of Pest Management, Sokoine University of Agriculture, Morogoro P.O. Box 67101, Tanzania; apiamas@yahoo.com (A.W.M.); rmakundi@yahoo.com (R.H.M.); 5 The Africa Centre of Excellence for Innovative Rodent Pest Management and Biosensor Technology Development, Sokoine University of Agriculture, Morogoro P.O. Box 67101, Tanzania; 6 Natural Resources Institute, University of Greenwich, Chatham Maritime, Kent ME4 4TB, UK; 7 Department of Wildlife, Fish & Environmental Studies, Swedish University of Agricultural Sciences, 75007 Umeå, Sweden

**Keywords:** rodent pest, fertility control, quinestrol, levonorgestrel, *Arvicanthis*

## Abstract

Rodent species belonging to the *Arvicanthis* genus cause substantial field and postharvest losses in many African countries, where management still relies mainly on lethal control methods. Hormonal fertility control may provide a complementary strategy for reducing rodent population growth. We investigated the antifertility effects of bait containing quinestrol, a synthetic estrogen, levonorgestrel, a synthetic progestogen, and their combination at 10, 50, and 100 ppm in the Nile grass rat (*Arvicanthis niloticus*). These compounds suppress fertility by disrupting normal reproductive endocrine function. Adult male and female animals were exposed to treated bait under laboratory conditions, and reproductive responses were evaluated through measurements of bait intake, body weight change, reproductive organ weights, sperm count, sperm motility, pregnancy rate, and litter size. Quinestrol and combined treatments significantly reduced male reproductive organ weights and impaired sperm quality. Female reproductive organ weights were generally unaffected, although uterine edema occurred at higher concentrations. Quinestrol at 50 ppm completely inhibited pregnancy in treated females, regardless of the male mating partner. In addition, untreated females paired with treated males exhibited lower pregnancy rates and reduced litter sizes. Our findings demonstrate the potential of hormonal fertility control as a complementary strategy for sustainable rodent pest management in Africa. Future studies should focus on improving bait intake, field evaluation, and determining the duration and reversibility of contraceptive effects.

## Simple Summary

Rodents are serious agricultural pests in many African countries because they damage crops in fields and storage facilities, leading to major food and economic losses. Current rodent control methods mainly rely on poisoning, trapping, sanitation of fields and storage areas, and hunting. However, these approaches are often insufficient for long-term population control and may also harm non-target species and the environment. Fertility control, which reduces the ability of animals to reproduce, may provide an additional and potentially more humane approach to rodent management. In this study, we tested whether bait containing two fertility regulating hormones could reduce reproduction in the Nile grass rat, an important African pest rodent species. The treated animals showed reduced reproductive performance, especially males, which had lower sperm production and sperm movement. Females treated with one of the hormones failed to become pregnant under certain treatment conditions. Some treated males also reduced pregnancy success when paired with untreated females. These findings show that fertility control may help reduce rodent populations when used together with existing rodent management methods. Further studies are needed to test how well this approach works under field conditions and whether the effects are reversible and safe for long-term use.

## 1. Introduction

Rodents are the most diverse and widely distributed group of mammals, occupying a broad range of habitats and exhibiting high reproductive potential and adaptive capacity [1,2,3]. Although they contribute to ecosystem functioning through seed dispersal, nutrient cycling, and serving as prey for predators [4,5], many rodent species are major agricultural pests. They damage crops at all stages of production, from cultivation to storage and across the entire food value chain, resulting in substantial economic losses. In addition, rodents contaminate food supplies and act as reservoirs for numerous zoonotic pathogens, with over 60 diseases reported to be transmitted to humans and livestock [6,7].

In sub-Saharan Africa, rodents are among the most destructive pests of cereal crops, causing substantial yield losses that vary depending on rodent population density, seasonal fluctuations, and outbreak dynamics [8,9,10]. During outbreak years, crop losses may exceed 80% in parts of East Africa [11]. In Ethiopia, rodent damage has been estimated to range from 9% to 44% of annual cereal production, with average losses of about 20% [12,13,14]. Among the major pest taxa in Africa, species of the genus *Arvicanthis* are particularly important because of their wide distribution, high reproductive capacity, and close association with agricultural landscapes. Several *Arvicanthis* species have been reported as serious pests of cereal crops across eastern, central, and western Africa, where they damage crops both before and after harvest. In Ethiopia, *A. niloticus* is one of the dominant rodent pests, causing damage to crops such as barley, wheat, maize, and teff [15,16]. In addition to their agricultural importance, *Arvicanthis* species may also serve as reservoirs of potentially zoonotic pathogens, thereby posing public health risks to humans [17,18].

Rodent management strategies in Africa have traditionally relied largely on lethal control methods, including rodenticides, trapping, and hunting [19,20]. Although these approaches can provide short-term reductions in rodent populations, repeated applications are often required because populations may recover rapidly through reproduction [21,22]. Consequently, fertility control has attracted increasing interest as a complementary management approach aimed at reducing population growth and enhancing the long-term effectiveness of integrated rodent management programs [23,24].

Fertility control does not aim to replace conventional rodent control methods but may serve as a complementary tool within Ecologically Based Rodent Management (EBRM) [25,26]. By reducing reproductive output and recruitment, fertility control has the potential to contribute to longer-term population suppression while reducing reliance on repeated lethal interventions [27,28,29]. However, the extent to which reductions in individual fertility translate into population level suppression depends on ecological and demographic processes operating under field conditions. Furthermore, because hormonal fertility control agents are endocrine active compounds, concerns regarding environmental persistence, non-target exposure, endocrine mediated ecological effects, and regulatory acceptability require careful evaluation before widespread implementation [30,31,32,33].

Fertility regulating agents act by interfering with key reproductive processes such as gametogenesis, endocrine regulation, ovulation, fertilization, and embryo implantation, with effects that may be either temporary or permanent depending on the compound and exposure level [34,35]. Despite its potential, the practical application of fertility control in African rodent pest management remains limited. Major challenges include ensuring bait palatability and species-specific uptake [29,36].

Among the fertility control agents investigated to date, steroid hormones such as quinestrol (QE), a synthetic estrogen, and levonorgestrel (LV), a synthetic progestogen, have shown considerable promise for managing rodent populations. These compounds are reported to disrupt reproductive endocrine pathways by inhibiting processes such as ovulation, follicular development, spermatogenesis, and embryo implantation [37,38]. Their combined formulation, commonly referred to as EP-1, has been evaluated in several rodent species and has demonstrated antifertility effects, including reductions in reproductive organ development, sperm production, and overall fertility rate [34,39,40]. Nevertheless, the response to hormonal fertility control agents varies considerably among rodent species, and their effectiveness is strongly influenced by factors such as dosage, duration of exposure, bait intake, and ecological context under which treatments are applied [29,41,42,43,44].

Although studies in Asia and Africa have demonstrated the potential of QE and LV for suppressing reproduction in several rodent pest species [28,34,39,43], information on their effects in *Arvicanthis* species remains very limited [45]. To our knowledge, only one previous study has investigated the effects of a fertility control agent in *A. niloticus*, demonstrating that QE affected reproductive organ development in this species [39]. However, no studies have comprehensively evaluated bait acceptance, physiological responses, reproductive performance, pregnancy rate, and litter size following exposure to QE, LV, or their combination (QL), and information on the effects of LV and QL in *A. niloticus* is lacking. This knowledge gap is particularly important given the wide distribution, high reproductive capacity, and major economic significance of *Arvicanthis* spp. in African agriculture. Because responses to fertility control agents are often species-specific, laboratory evaluation in target pest species is an essential prerequisite for subsequent demographic modelling, environmental risk assessment, and field implementation.

Responses to fertility control agents vary largely among rodent species. In Brandt’s voles, *Lasiopodomys brandtii*, QE caused hepatic toxicity and pronounced reproductive effects [42], whereas in plateau zokors, *Eospalax baileyi*, LV showed little reproductive efficacy while QE induced strong physiological changes [43]. Bait palatability also differed among species, with reduced consumption reported in *Mastomys natalensis* and *Rattus rattus* [39,44] and particularly poor acceptance in *R. argentiventer* [40]. These interspecific differences highlight the need for species-specific evaluation before fertility control agents can be applied in pest management programs. Accordingly, the present study aimed to assess the effects of bait containing QE, LV, and their combination (QL) at concentrations of 10, 50, and 100 ppm on *A. niloticus*. Specifically, we evaluated bait intake, physiological responses, and reproductive performance following contraceptive bait consumption under controlled laboratory conditions.

## 2. Materials and Methods

### 2.1. Study Area and Experimental Animals

The study was conducted at the Rodent Research Unit Laboratory in the Department of Biology, Mekelle University. Adult *A. niloticus* were live trapped from agricultural fields within the university campus using standard Sherman live traps (23 × 9.5 × 8 cm; H.B. Sherman Traps Inc., Tallahassee, FL, USA) baited with peanut butter. Captured animals were transferred carefully into individual cages using zippered plastic bags to minimize handling stress.

Animals were housed individually in cages (20 × 20 × 30 cm) under controlled laboratory conditions (12:12 h light–dark photoperiod, 20–30 °C ambient temperature, and 40–60% relative humidity). Animals were acclimatized for 10 days prior to experimentation. During acclimatization, standard commercial pellets and water were provided *ad libitum*, and animals were monitored daily for health status, activity, feeding behavior, and general condition. Pregnant, unhealthy, or injured individuals were excluded from the study. No mortality occurred during the acclimatization period. Only females with a closed vagina (i.e., non-receptive and not recently mated) were included in the experiment. A total of 160 adult individuals (80 males and 80 females) were finally used for the experiment. Mean body weights at the start of the study were 100.67 g for males and 85.56 g for females.

### 2.2. Bait Preparation and Formulation

Contraceptive bait preparation followed previously described protocols e.g., [39]. Briefly, QE and LV obtained from Beijing Zizhutiangong Science and Technology Ltd., Beijing, China, were formulated at concentrations of 10, 50, and 100 ppm. Three bait formulations were prepared: QE alone, LV alone, and a combined formulation (QL) containing QE and LV at a 1:1 ratio. Each contraceptive compound was dissolved in 100 mL absolute ethanol using a water bath (maintained at 60–70 °C), then mixed with a sucrose solution (prepared by dissolving 200 g sucrose in 1000 mL distilled water) to produce a homogeneous milky suspension. The suspension was continuously stirred and uniformly sprayed onto standard bait to ensure even coating of the active ingredients. Control bait (0 ppm) was prepared using the same procedure but without the addition of hormonal contraceptives.

### 2.3. Bait Intake and Body Weight Assessment

A total of 120 animals were used to evaluate bait intake, with each treatment group consisting of five animals of both sexes. Animals were provided with bait equivalent to 10% of their body weight, with treatment durations of seven days for females and 14 days for males, while water was supplied ad libitum. The longer exposure period for males was based on previous studies demonstrating that extended treatment durations are generally required to achieve effective suppression of spermatogenesis and fertility in male rodents, whereas shorter exposure periods are often sufficient to disrupt female reproductive function [46,47]. Daily bait intake was determined by subtracting the remaining bait weight from the initial amount provided and expressed as a percentage using the formula: percent consumption = [(initial bait weight − remaining bait weight)/initial bait weight] × 100. Body weight of each individual was recorded at the start of the experiment and monitored daily throughout the treatment period. Total weight change was calculated as the difference between body weight at the start of treatment (X) and at the end of treatment (Y). Percentage body weight change was calculated as [(Y − X)/X] × 100.

### 2.4. Assessment of Reproductive Physiology

Animals were euthanized on day 8 for females and day 15 for males following established guidelines [48], using cervical dislocation after anesthesia. The different termination times corresponded to the sex-specific treatment durations applied in the experiment, with males maintained for a longer exposure period to allow adequate assessment of contraceptive effects on spermatogenesis and associated reproductive parameters. Reproductive organs were carefully dissected and weighed, including the uterus and ovaries in females, and the testes, epididymis, and seminal vesicles in males. Sperm analysis was conducted according to established protocols [39,49]. Briefly, the caudal epididymis was dissected and placed in 1 mL of 0.9% normal saline to obtain a sperm suspension. Sperm count was determined using a hemocytometer, while motility and morphology were assessed from prepared smears stained with nigrosine-eosin. All samples were processed under standardized laboratory conditions and examined microscopically at ×100 magnification.

### 2.5. Reproductive Performance (Pregnancy and Litter Size)

Based on preliminary findings, QE at 50 ppm was selected for reproductive performance assessment due to its relatively stronger antifertility effects. Although both QE and LQ at 50 ppm demonstrated substantial reductions in reproductive parameters, QE showed slightly higher effects and was therefore selected for subsequent pregnancy and litter size assessment. At a higher dose of 100 ppm, some of the rats exhibited signs of illness, and further pregnancy and litter size were not conducted. A total of 40 adult *A. niloticus* (20 males and 20 females) were used for this experiment. Females were exposed to treatment for 7 days and males for 14 days prior to pairing. Animals were then paired for 10 days in four mating combinations: untreated female × untreated male (UF × UM, control), untreated female × treated male (UF × TM), treated female × untreated male (TF × UM), and treated female × treated male (TF × TM), with five females included per group. Following pairing, females were maintained on untreated bait for 30 days, during which pregnancy and litter size weight were recorded.

### 2.6. Data Analysis

All statistical analyses were done using SPSS version 29 (IBM Corp., Armonk, NY, USA). Prior to analysis, data were examined for compliance with the assumptions of parametric tests. Normality of the data distribution was assessed using the Kolmogorov-Smirnov test, while homogeneity of variances was evaluated using Levene’s test.

For bait intake, body weight, reproductive organ weights, sperm parameters (count and motility), and litter size, differences among treatment groups were analyzed using one-way analysis of variance (ANOVA). When significant differences were detected, Tukey’s honestly significant difference (HSD) post hoc test was applied for pairwise comparisons among treatment groups. Bait intake was expressed as percentage consumed relative to the initial amount offered. Changes in body weight were calculated both as absolute change (g) and percentage change relative to initial body weight. Reproductive parameters, including organ weights and sperm characteristics, were expressed as mean ± standard error (SE).

Pregnancy rate was calculated as the proportion of pregnant females within each treatment group and expressed as a percentage. Differences in mean litter size among mating combinations were analyzed using one-way ANOVA, followed by Tukey’s HSD test for pairwise comparisons. All statistical tests were two-tailed, and differences were considered statistically significant at *p* < 0.05. Results are presented as mean ± SE unless otherwise stated.

### 2.7. Ethical Compliance

All procedures involving animal handling were conducted in accordance with internationally accepted guidelines for the care and use of laboratory animals and were reviewed and formally approved by the Animal Ethics and Experimentation Committee (AEEC No: AEEC01/2025) of Mekelle University, Ethiopia, prior to the commencement of the study. Field trapping, animal handling, transportation, and acclimatization procedures were conducted under controlled conditions following established standard procedures with continuous health monitoring to ensure animal welfare. All experimental procedures involving feeding trials, body weight monitoring, and reproductive assessments were designed to minimize distress. The number of animals used was kept to the minimum required to achieve statistical validity, and animals were housed individually under appropriate environmental conditions with food and water provided ad libitum. Euthanasia procedures were performed in accordance with the American Veterinary Medical Association (AVMA) Guidelines for the Euthanasia of Animals [48], which are widely recognized as the international standard for humane endpoints in laboratory research. All reproductive organ collection and sperm analyses were conducted postmortem. Laboratory analyses, including sperm count and morphology assessments, followed standardized protocols [48,49].

## 3. Results

### 3.1. Bait Intake

**Figure 1:**
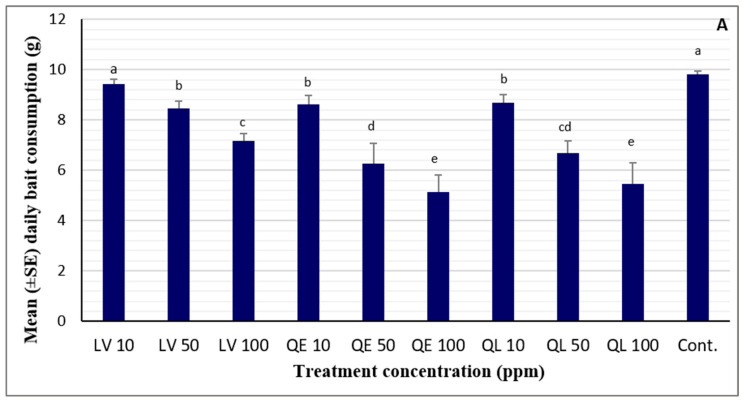

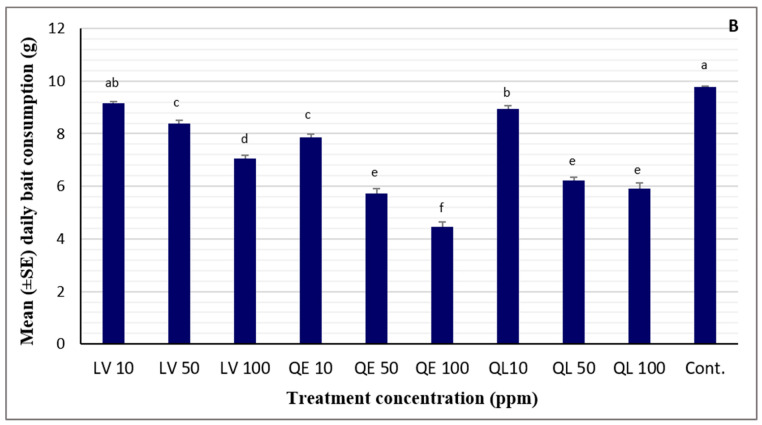
Mean (±SE) daily bait intake of adult female (**A**) and male (**B**) *Arvicanthis niloticus* exposed to contraceptive treated bait (levonorgestrel (LV), quinestrol (QE), and their combination (QL)) at three concentrations (10, 50, and 100 ppm). Different letters indicate statistically significant differences among treatments (*p* < 0.05), whereas shared letters indicate no significant difference. Control animals (Cont.) received untreated bait.

**Figure 2:**
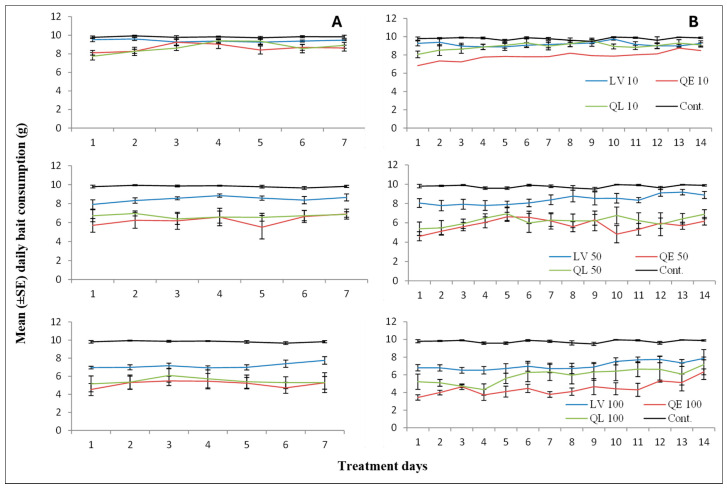
Mean (±SE) daily bait intake of adult female (**A**) and male (**B**) *Arvicanthis niloticus* exposed to bait formulations containing LV, QE, and their combination (QL) at concentrations of 10, 50, and 100 ppm. Control animals (Cont.) received untreated bait.

Bait intake was significantly reduced in both female (F = 152.57, df = 9, *p* < 0.0001) and male (F = 156.95, df = 9, *p* < 0.0001) *A. niloticus* exposed to contraceptive treatments compared with the control group, except in those treated with LV at 10 ppm (Figure 1A,B). Among treatments, QE and QL produced the highest reduction in bait intake, particularly at higher concentrations (50 and 100 ppm), where differences were highly significant (*p* < 0.0001) for both sexes. In contrast, LV had a comparatively weaker effect on bait intake (i.e., relatively high intake). Sex-specific differences were also observed, with males consistently consuming less bait than females in most treatments (Figure 1A,B). Despite the overall reduction, mean daily bait intake showed a gradual increase over the exposure period for both sexes (Figure 2).

### 3.2. Body Weight

**Figure 3:**
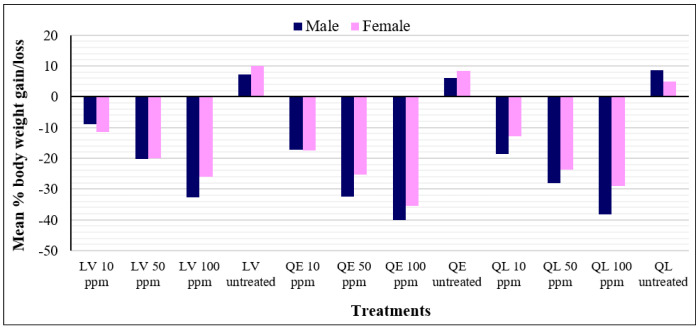
Percentage change in body weight of *Arvicanthis niloticus* following consumption of bait containing LV, QE, and QL at different concentrations. Data are presented as mean ± standard error (SE). The value “0” on the Y-axis represents no change in body weight relative to the initial body weight. Positive values indicate body weight gain, whereas negative values indicate body weight loss during the treatment period.

Body weight responses varied among treatments, concentrations, and sexes (Figure 3). Overall, animals exposed to contraceptive bait showed reductions in body weight relative to control groups, with the magnitude of weight loss generally increasing with increasing bait concentration. In contrast, control groups showed moderate body weight gain during the experimental period.

Among the treatments, QE at 100 ppm produced the greatest reduction in body weight in both sexes, followed by QL at 100 ppm. Male animals generally exhibited greater body weight loss than females across most treatment groups.

Overall, the results indicate a dose dependent effect of contraceptive bait consumption on body weight, particularly in animals treated with QE and QL formulations.

### 3.3. Reproductive Physiology 

**Figure 4:**
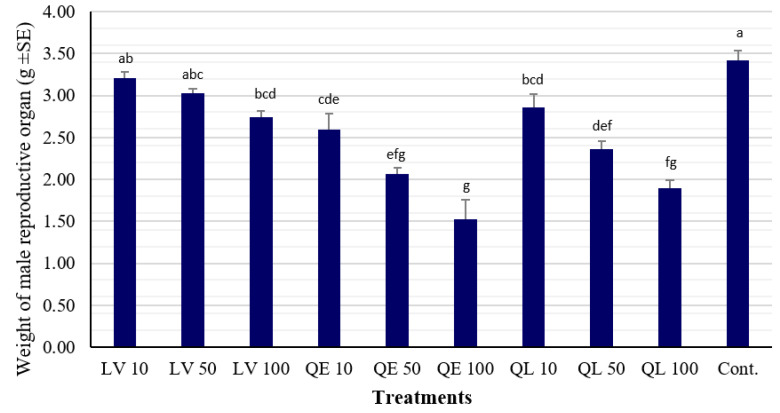
Mean (±SE) weight of reproductive organs of treated and control male *Arvicanthis niloticus*. Different superscript letters indicate significant differences among treatments (*p* < 0.05; Tukey’s HSD test).

Significant differences were observed in the mean weight of male reproductive organs (testes, epididymis, and seminal vesicles) among treatments (F_9,40_ = 21.952, *p* < 0.001; Figure 4). Reductions in organ weight were recorded in all hormone treated groups compared to the control, except for LV at 10 and 50 ppm. The magnitude of reduction increased with concentration, with the lowest mean organ weights recorded in QE 100 ppm, QL 100 ppm, and QE 50 ppm treatments.

Sperm count differed significantly among treatments (F_9,40_ = 21.448, *p* < 0.001; Table 1). All treated groups exhibited reduced sperm counts relative to the control. The lowest sperm counts were recorded in QE 50 ppm, QE 100 ppm, and QL 100 ppm treatments. Sperm motility also varied significantly among treatments (F_9,40_ = 48.238, *p* < 0.001; Table 1). All hormone treated groups showed lower sperm motility compared to the control group. The lowest motility values were observed at QE 100 ppm (36.80%) and QE 50 ppm (43.60%). The proportion of non-viable sperm cells was higher in the treated groups compared to the control.

**Figure 5:**
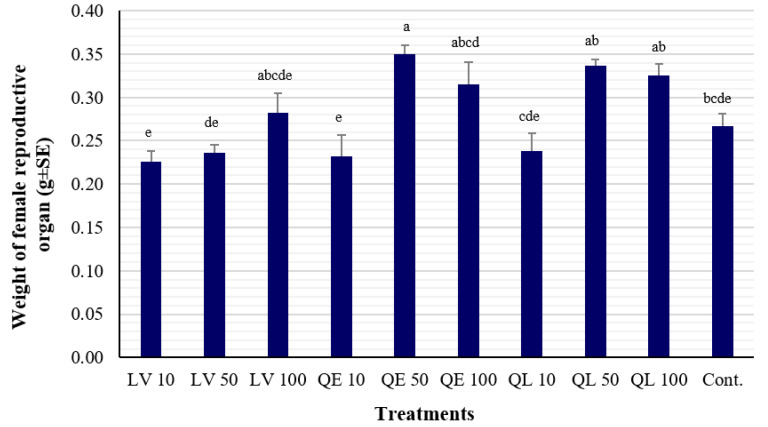
Mean (±SE) weight of reproductive organ of treated and control female *Arvicanthis niloticus*. Different superscript letters indicate significant differences among treatments (*p* < 0.05; Tukey’s HSD test).

Significant differences were also observed in the mean weight of female reproductive organs (uterus and ovaries) among treatments (F_9,40_ = 7.92, *p* < 0.001; Figure 5). Overall, female reproductive organ weights tended to increase with increasing hormone concentration. A significantly higher mean reproductive organ weight was recorded in the QE 50 ppm treatment compared to the control (*p* < 0.05), while other treatments did not differ significantly from the control.

### 3.4. Reproductive Performance

The effect of QE at 50 ppm on pregnancy and litter size was evaluated across different mating combinations. Significant differences were observed in mean litter size among pairing groups (F_3,16_ = 220.00, *p* < 0.001).

The control group (untreated female × untreated male) showed an 80% pregnancy rate, with a mean litter size of 7.00 ± 0.32 pups. Pairing untreated females with treated males resulted in a reduced pregnancy rate of 40% and a mean litter size of 3.00 ± 0.32 pups. No pregnancies were recorded in groups where treated females were paired with either untreated or treated males (Table 2).

## 4. Discussion

This study evaluated, for the first time, the effects of the fertility limiting hormones QE, LV, and their combination Q) on bait intake, physiological condition, and reproductive performance of the Nile grass rat (*A. niloticus*), a major agricultural rodent pest in Africa. Under laboratory conditions, both individual and combined hormone treatments affected bait consumption, body weight, reproductive organ characteristics, sperm quality, pregnancy rate, and litter size, although the magnitude of these effects varied among compounds and concentrations. The strongest antifertility effects were observed in animals exposed to QE containing baits, particularly at 50 ppm, which completely prevented pregnancy in treated females. These findings provide the first species-specific evidence that hormonal fertility control agents can substantially impair reproduction in *A. niloticus* and establish a foundation for future studies examining their potential role within integrated rodent pest management strategies. However, because the present study was conducted under controlled laboratory conditions, the implications of these reproductive effects for population suppression, environmental safety, and practical field implementation require further investigation.

### 4.1. Effects on Bait Intake

A consistent reduction in bait consumption was observed in most of the hormone treated groups, particularly at 50 and 100 ppm for QE and QL in both sexes. Reduced bait intake following exposure to hormonal contraceptives has been reported in several rodent species, in *Mastomys natalensis* [39], *Rattus rattus* [44], and *R. argentiventer* [40], and is generally attributed to alterations in palatability, metabolic feedback, or endocrine mediated changes in feeding regulation [29,36,50]. Steroidal compounds, particularly estrogenic agents such as QE, can influence hypothalamic pathways involved in appetite regulation, potentially reducing feeding motivation [51,52]. However, not all studies report such effects. For example, no significant influence of QE on bait consumption was found in Brandt’s voles *Lasiopodomys brandtii* [53], highlighting interspecific variability in behavioral and physiological responses to contraceptive compounds.

The relatively higher intake of LV treated bait compared to QE and QL suggests compound-specific differences in taste perception or physiological tolerance. Relatively, female *A. niloticus* consumed slightly more bait than males, suggesting sex related variation in feeding response. From an applied perspective, reduced bait intake at higher concentrations remains a key limitation for field deployment of fertility control agents in rodent management programs in agroecosystems [36,54]. Consequently, optimizing bait formulation to maximize palatability while maintaining contraceptive effectiveness will be an important consideration for future development of fertility control strategies targeting *A. niloticus*.

### 4.2. Effects on Body Weight and Physiological Condition

Both male and female rats in treated groups exhibited significant body weight loss, whereas control animals gained slight weight (Figure 3), indicating that hormonal exposure negatively affected physiological condition. This pattern likely reflects a combined influence of reduced food intake and endocrine mediated alterations in energy balance. Comparable reductions in body weight have been reported in multiple rodent species exposed to QE or combined hormonal treatments [39,40,55], and other rodent fertility control studies using steroidal compounds, including *Meriones unguiculatus*, *L. brandtii*, and *R. argentiventer* [38,40,55,56].

The magnitude of weight loss varied across treatments, with stronger effects observed at higher concentrations of QE and QL. Estrogenic compounds such as QE are known to influence appetite regulation, lipid metabolism, and overall energy homeostasis, which may contribute to reduced body mass [34,57]. However, some studies have reported limited or inconsistent effects on body weight under similar treatments [34,44]. These discrepancies highlight the importance of species-specific responses, as well as differences in dosage, exposure duration, and experimental design. However, untreated groups showed slight weight gain over time, confirming that laboratory conditions supported normal growth in the absence of hormonal exposure.

### 4.3. Effects on Reproductive Physiology

Significant reductions in reproductive organ weight, sperm count, and sperm motility were observed in treated males, particularly under QE and higher doses of QL treatments (Figure 4, Table 1). The reduction in sperm quality aligns with known mechanisms of estrogenic and progestogenic compounds, which can suppress the hypothalamic-pituitary-gonadal (HPG) axis [37,58]. QE is known to inhibit gonadotropin releasing hormone (GnRH), thereby reducing downstream follicle stimulating hormone (FSH) and luteinizing hormone (LH), ultimately impairing spermatogenesis [55]. LV may further contribute by interfering with steroid feedback loops regulating testicular function [38].

The pronounced decline in sperm motility and increased presence of dead sperm cells indicate not only reduced sperm production but also impaired sperm viability, consistent with findings reported in *R. argentiventer*, *M. natalensis*, and *O. curzoniae* exposed to similar compounds [34,39,40]. These combined effects strongly support the antifertility potential of these compounds in male *A. niloticus*.

In females, reproductive organ weights were less consistently affected, although significant increases in uterine weight were observed at higher QE concentrations (Figure 5). This increase is most likely attributable to uterine oedema rather than enhanced reproductive function. Estrogenic overstimulation is known to induce fluid accumulation, endometrial thickening, and disruption of normal reproductive cycles [59]. The absence of consistent ovarian weight changes suggests that hormonal effects may primarily disrupt reproductive function at the level of implantation and uterine receptivity rather than ovarian structure [60,61].

### 4.4. Effects on Reproductive Success (Pregnancy and Litter Size)

QE at 50 ppm produced a complete inhibition of pregnancy in treated females regardless of male treatment status, indicating strong contraceptive efficacy in females (Table 2). In contrast, untreated females mated with treated males exhibited reduced pregnancy rates and smaller litter sizes, indicating partial male mediated fertility suppression. The absence of pregnancy in treated females suggests disruption at multiple reproductive stages, including ovulation inhibition, altered uterine receptivity, and impaired implantation, as previously documented in rodent models exposed to QE based treatments [55,62]. Uterine edema observed in this study may further contribute by physically impairing implantation [59]. The reduction in litter size in untreated females paired with treated males further confirms reduced male fertility due to compromised sperm quality, consistent with studies in *R. nitidus* and *R. rattus* [42,58].

### 4.5. Implications for Rodent Population Management

The present study provides the first evaluation of hormonal fertility control agents in *A. niloticus*, an important agricultural rodent pest throughout sub-Saharan Africa. Although QE and LV have previously demonstrated antifertility effects in several rodent species [34,38,40,55,56], responses to contraceptive agents often vary among species because of differences in reproductive physiology, life-history traits, feeding ecology, and hormone sensitivity [29]. Consequently, species-specific assessments are essential before fertility control can be considered a viable management option.

The substantial reductions in sperm quality, alterations in reproductive organ characteristics, complete suppression of pregnancy in females exposed to QE containing treatments, and reductions in litter size observed in this study demonstrate that *A. niloticus* is highly responsive to hormonal fertility control agents under laboratory conditions. These findings suggest that fertility control could potentially reduce reproductive output and recruitment, key demographic processes underlying population growth in many rodent pest species [29,30,36].

However, translating individual level reproductive impairment into population level suppression is unlikely to be straightforward. The effectiveness of fertility control depends on several ecological and operational factors, including bait acceptance, treatment coverage, population density, survival rates, immigration from surrounding populations, and compensatory demographic responses [29,30]. In highly fecund rodent species, reductions in reproduction among treated individuals may be partially offset by increased survival, enhanced breeding success among untreated animals, or recolonization from neighboring populations. Consequently, substantial reductions in fertility do not necessarily result in proportional reductions in population size or crop damage [54].

Nevertheless, the complete suppression of pregnancy observed in females receiving QE containing treatments and the clear reductions in litter production across treatment groups indicate that hormonal fertility control warrants further investigation as a component of integrated rodent management strategies for *A. niloticus*. Future studies combining field experiments with demographic modelling will be required to determine whether the reproductive effects observed under laboratory conditions can generate meaningful reductions in population abundance and agricultural losses under operational conditions.

### 4.6. Environmental Considerations and Regulatory Implications

Although fertility control is often considered as a more humane alternative or complement to conventional lethal rodent management, hormonal contraceptive agents are not inherently free of environmental risks. QE and LV are endocrine active compounds that achieve contraceptive effects by disrupting normal reproductive physiology. While this mode of action is desirable in target species, unintended exposure of non-target organisms could potentially result in adverse endocrine mediated effects [31,32,33,63,64].

Environmental exposure pathways, environmental persistence, and potential non-target exposure therefore require careful evaluation before field deployment; see [29,30,63,65]. Steroidal hormones may enter terrestrial and aquatic ecosystems through bait residues, excreta, carcasses, or environmental transport processes [63,66,67,68,69]. Exposure to endocrine active compounds has been shown to affect reproduction, development, and population dynamics in a range of non-target organisms, particularly aquatic vertebrates such as fish and amphibians [31,32,33]. Consequently, assessments of environmental persistence, residue dynamics, and non-target exposure pathways are essential components of fertility control risk evaluation [29,35,63].

Available evidence suggests that both QE and LV exhibit relatively limited persistence in soil. QE has been reported to degrade in soil with a half-life of ~15–16 days and undergo rapid photodegradation in water, with half-lives of less than three hours under visible or ultraviolet light exposure [66,67,68]. Similarly, LV exhibits soil half-lives ranging from approximately 4 to 12 days [66,67,68]. However, persistence may increase substantially in aquatic environments with limited light penetration, where QE residues have been reported to remain detectable for 60–90 days, thereby increasing the potential for exposure of aquatic organisms and associated endocrine disrupting effects [66,69]. Current evidence also suggests that risks to terrestrial non-target vertebrates, including birds, may be minimized through appropriate bait formulation and delivery systems, although species-specific ecological risk assessments remain necessary before operational use [65,70].

Beyond environmental considerations, regulatory approval of fertility-control technologies may represent an additional challenge for the implementation. Any future registration and field deployment of QE or LV based baits would require thorough environmental risk assessment, evaluation of non-target impacts, residue studies, and compliance with national regulatory frameworks governing pesticide and wildlife management products. Accordingly, the development of effective bait delivery systems that minimize exposure of non-target species should be an important component of future research and product development.

### 4.7. Limitations and Future Directions

Several limitations should be considered when interpreting the findings of this study. First, the experiment was conducted under controlled laboratory conditions that may not fully reflect feeding behavior, bait selection, environmental variability, social interactions, and alternative food availability encountered by wild rodent populations. Consequently, treatment efficacy observed under laboratory conditions may differ from that achieved in the field.

Second, reproductive responses were assessed over a relatively short period, and the persistence and reversibility of contraceptive effects were not evaluated. Long-term studies are therefore needed to determine the duration of fertility suppression and the extent to which reproductive function recovers following cessation of treatment.

Third, environmental fate, residue dynamics, and non-target exposure were beyond the scope of the present study. Because these factors are critical for evaluating the ecological safety and regulatory acceptability of hormonal fertility control agents, they should be incorporated into future research.

Future research should therefore focus on validating the efficacy of these compounds under field conditions [29,36,65], optimizing bait formulation and delivery systems to improve palatability and target specificity, assessing the duration and reversibility of contraceptive effects, and evaluating environmental persistence and non-target risks [25,71]. In addition, demographic modelling and field scale experiments will be essential to determine whether the reductions in fertility observed here can translate into meaningful suppression of *A. niloticus* populations and associated agricultural damage.

## 5. Conclusions

QE, either alone or in combination with LV, significantly reduced reproductive performance in *A. niloticus* under laboratory conditions. Treatment effects included reductions in reproductive organ weights, impaired sperm quality, complete suppression of pregnancy in females exposed to QE containing baits, and significant reductions in litter production. These findings provide the first species-specific evidence that hormonal fertility control has potential as a complementary component of integrated management strategies for this important African agricultural rodent pest. However, laboratory efficacy alone is insufficient to predict operational success. Further research is required to evaluate field performance, population level consequences, environmental safety, non-target effects, and regulatory feasibility before hormonal fertility control agents can be considered for practical implementation in rodent management programs.

## Figures and Tables

**Table 1:** Sperm parameters of *Arvicanthis niloticus* following exposure to contraceptive hormones.

Treatment (ppm)	Sperm Count (×10^6^)	Sperm Motility (%)
LV 10	65.28 ± 6.48 ^b^	75.50 ± 1.75 ^b^
LV 50	54.56 ± 3.90 ^bcd^	67.50 ± 1.72 ^bc^
LV 100	46.70 ± 0.99 ^bcd^	64.00 ± 1.37 ^cd^
QE 10	29.98 ± 5.35 ^bcd^	56.20 ± 2.34 ^d^
QE 50	18.72 ± 0.62 ^cd^	43.60 ± 3.08 ^e^
QE 100	17.36 ± 0.48 ^cd^	36.80 ± 2.40 ^e^
QL 10	38.70 ± 4.13 ^bcd^	62.50 ± 1.78 ^cd^
QL 50	26.16 ± 4.99 ^bcd^	54.50 ± 2.42 ^d^
QL 100	19.50 ± 3.13 ^cd^	55.20 ± 2.00 ^d^
Control	161.99 ± 27.07 ^a^	87.60 ± 1.84 ^a^

Note: Values are presented as mean ± SE. Different superscript letters within a column indicate significant differences among treatments (*p* < 0.05; Tukey’s HSD test).

**Table 2:** The effects of QE (50 ppm) on pregnancy and litter size of *Arvicanthis niloticus*. UF = untreated female; TF = treated female; UM = untreated male; TM = treated male.

Pairing Combination	No. of Pregnant Females (*n* = 5/Pairing)	Pregnancy Rate (%)	Mean Litter Size
UF × UM	4	80	7.00 ± 0.32 ^a^
UF × TM	2	40	3.00 ± 0.32 ^b^
TF × UM	0	0	0.00 ± 0.00 ^c^
TF × TM	0	0	0.00 ± 0.00 ^c^

Note: Values are presented as mean ± SE. Different superscript letters indicate significant differences among treatments (*p* < 0.05; Tukey’s HSD test).

## Data Availability

The data presented in this study are available upon request from the corresponding authors.

## Abbreviations

The following abbreviations are used in this manuscript:

QE	Quinestrol
LV	Levonorgestrel
QL	Quinestrol and Levonorgestrel combination
PPM	Parts Per Million
EBRM	Ecologically Based Rodent Management
